# Antigenic structural analysis of bat and human norovirus protruding (P) domains

**DOI:** 10.1128/jvi.01971-24

**Published:** 2025-03-10

**Authors:** Dayna L. Holroyd, Akhil Kumar, Eduardo Vasquez, Veronika Masic, Mark von Itzstein, John B. Bruning, Grant S. Hansman

**Affiliations:** 1 Institute of Photonics and Advanced Sensing (IPAS), School of Biological Sciences, The University of Adelaide 613297 , Adelaide, South Australia, Australia; 2 Institute for Biomedicine and Glycomics (IBG), Griffith University, Gold Coast Campus, Gold Coast, Queensland, Australia; St. Jude Children's Research Hospital, Memphis, Tennessee, USA

**Keywords:** noroviruses, X-ray crystallography, antigenic variation

## LETTER

Noroviruses are highly contagious, infecting both humans and animals. At least 10 genogroups exist (GI–GX), which are further divided into genotypes with the GII genotypes causing most outbreaks (1). The human norovirus capsid protruding (P) domain binds histo-blood group antigen (HBGA) co-factors for infection (2, 3), and a recently discovered bat norovirus (GX) was also found to interact with HBGAs (4). The genetic variability of the capsid gene is coupled with antigenic diversity and associated with escape from herd immunity, emergence of antigenic variants, and possible changes in host tropism (4–13). In this study, we determined the P domain structures of four human noroviruses and one bat norovirus using X-ray crystallography to examine structural features linked to potential interspecies transmission.

**Fig 1:**
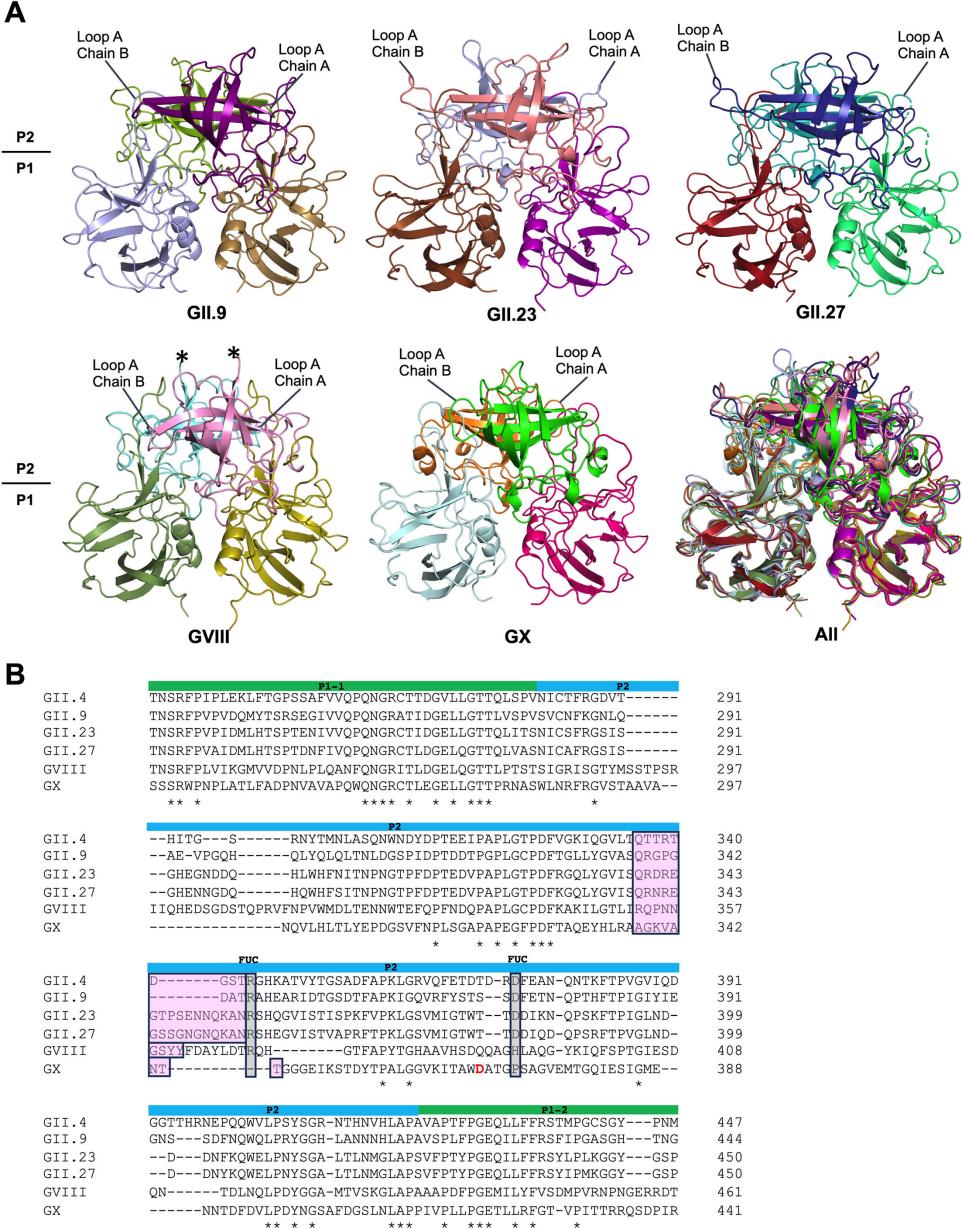
Four human and one bat norovirus X-ray crystal structures and a corresponding P domain sequence alignment. (**A**) The P domains of GII.9, GII.23, GII.27, GVIII, and GX are shown in cartoon representation. The asterisks indicated missing residues in the structure, likely due to the flexibility of the loop. The P domain, subdivided into P1 and P2 subdomains, is colored chain A P1/chain B P1 and chain A P2/chain B P2 for GII.9 (sand/ light blue and deep purple/split pea), GII.23 (purple/ brown and salmon/ light blue), GII.27 (lime green/ firebrick and deep blue/ teal), GVIII (olive/ smudge and pink/ aquamarine), and GX (hot pink/ pale cyan and green/ orange), respectively. Loop A is located near the HBGA pocket and commonly contains the essential fucose moiety binding residue (e.g., GII.4 R345) and is longer in GII.23 (~17 residues) and GII.27 (~17 residues) than in GII.9 (~9 residues), GVIII (~9 residues), and GX (~8 residues). (**B**) Clustal W sequence alignment showing the partial P domains of GII.9, GII.23, GII.27, GVIII, GX, and with consensus GII.4 (Syd-2012, JX459908). The P2 subdomains contain numerous insertions and deletions compared to the P1 subdomains. Loop A (pink highlight) and two regular HBGA binding residues in GII.4 P domains (R345 and D374, gray highlight, and termed FUC) are shown. The two equivalent residues in the GII.9 (R346 and D374), GII.23 (R355 and D384), and GII.27 (R355 and D384) P domain structures are positioned similarly to the GII.4 P domain. The GVIII and GX P domains lack these two residues, except for GX D367 (highlighted red), which is located on the equivalent loop and near GII.4 D374.

The P domains of GII.9 (AY038599), GII.23 (KT290889), GII.27 (MG495077), GVIII (AJ844470), and GX (MF373609) were expressed in *E.coli* (14). Protein crystals were grown using a sitting drop method (Table 1) and X-ray diffraction was collected as described (15, 16). The five P domains were structurally equivalent with a root mean square deviation for C-alpha atoms ranging between 0.586 Å (GII.23-GII.27) and 1.645 Å (GII.9-GX). The principal structural difference was a variable length P2 subdomain loop (Loop A), which was located near the HBGA pocket (Fig. 1A). Two P domain residues that regularly bind HBGAs (e.g., GII.4 R345 and D374) were conserved in GII.9, GII.23, and GII.27 P domains (Fig. 1B). Also, these residue side chains were properly positioned to bind the fucose moiety of HBGAs (structure not shown). These two equivalent residues were lacking in the GVIII and GX P domains; however, an aspartic acid (D367) was located nearby in the GX P domain (Fig. 1B).

**TABLE 1:** Data collection and refinement statistics of P domain X-ray crystal structures

	GII.9	GII.23	GII.27	GVIII	GX
**Data collection**					
Space group	P 21 21 2	P 1 21 1	P 1 21 1	P 31 2 1	P 32 2 1
Cell dimensions					
*a*, *b*, *c* (Å)	94.2 96.9 65.8	60.1 81.0 71.8	51.2 90.6 67.1	107.2 107.2 61.6	67.5 67.5 238.8
*α, β, γ* (°)	90.0 90.0 90.0	90.0 113.4 90.0	90.0 106.3 90.0	90.0 90.0 120.0	90.0 90.0 120.0
Resolution range (Å)	48.44–1.98 (2.03–1.98)	45.59–1.22 (1.24–1.22)	49.09–1.42 (1.45–1.42)	46.44–1.76 (1.79–1.76)	47.75–1.63 (1.66–1.63)
No. unique reflections	42429 (2901)	185179 (8742)	109595 (4942)	40834 (2291)	79623 (12627)
*R*_merge_^*a*^	0.226 (1.766)	0.142 (0.626)	0.086 (0.684)	0.104 (0.992)	0.143 (1.626)
*R*_meas_*^b^*	0.237 (1.848)	0.165 (0.717)	0.094 (0.750)	0.108 (1.030)	0.149 (1.691)
*R*_pim_^*c*^	0.070 (0.541)	0.083 (0.344)	0.039 (0.305)	0.029 (0.273)	0.040 (0.458)
<I/σ(I)>	11.75 (1.8)	9.7 (3.6)	12.0 (2.8)	15.4 (1.87)	14.3 (1.8)
CC_1/2_	0.996 (0.573)	0.986 (0.774)	0.998 (0.870)	0.999 (0.843)	0.999 (0.643)
Completeness	99.5 (98.2)	99.2 (94.9)	99.5 (90.7)	99.9 (99.0)	99.9 (97.2)
Multiplicity	11.3 (11.3)	3.8 (3.8)	5.7 (5.6)	13.8 (13.6)	13.5 (13.4)
**Refinement**					
Resolution range (Å)	47.13–1.98 (2.03–1.98)	40.48–1.22 (1.23–1.22)	49.08–1.42 (1.44–1.42)	46.44–1.76 (1.80–1.76)	47.11–1.63 (1.65–1.63)
*R*_work_^*d*^	0.1645 (0.2543)	0.1226 (0.1585)	0.1316 (0.1919)	0.1395 (0.2201)	0.1624 (0.2421)
*R*_free_*^d^*	0.2095 (0.3041)	0.1463 (0.1997)	0.1720 (0.2540)	0.1807 (0.2591)	0.1938 (0.2325)
No. atoms	5284	11721	5987	3250	5682
Protein	4725	10203	5026	2584	4782
Water	557	1345	960	665	880
Ligand	2	173	1	1	20
B-factors (Å^2^)	27.37	14.69	25.39	24.13	22.45
Protein	26.7	10.67	22.61	20.47	19.96
Water	32.91	29.23	39.93	38.33	35.32
Ligand	66.74	29.32	25.78	28.06	51.15
RMS bond length (Å)	0.006	0.007	0.005	0.006	0.006
RMS bond angle (°)	0.83	1	0.77	0.82	0.8
**Ramachandran plot statistics** ^*e*^					
Residues	605	619	614	305	594
Most favored region	98.67	97.38	96.7	96.35	98.47
Allowed region	1.16	2.62	3.3	3.32	1.53
Disallowed region	0.17	0	0	0.33	0
Clashscore	2.27	2.6	1.22	2.52	2.12
**PDB ID**	9EDM	9EDN	9EDO	9EDP	9EDQ
**Mother solution**	0.1 M HEPES pH 7.0, 30% vol/vol Jeffamine ED-2003	0.1 M sodium acetate (pH 4.6), 8% wt/vol PEG 4000	0.17 M ammonium sulfate, 25.5% wt/vol PEG 4000	0.1 M Bis-Tris (pH 5.5), 25% wt/vol PEG 3350	1.6 M magnesium sulfate heptahydrate, 0.1 M MES (pH 6.5)

^*a*^ *R*_merge_ = Σ_h_ Σ_i_ |*I*_(h)i_ – *I*_h_| / Σ_h_ Σ_i_ *I*_(h)i_ where *I*_h_ is the averaged intensity of all reflections *h*.

^*b*^ *R*_meas_ = Σ_h_ [*N*/ (*N* – 1)]^1/2^ Σ_i_| *I*_(ih)_ – *I*_h_ |/Σ_h_ Σ_i_ *I*_(ih)_.

^*c*^ *R*_pim_ = Σ_h_ [1/ (*N* – 1)]^1/2^ Σ_i_| *I*_(ih)_ – *I*_h_ |/Σ_h_ Σ_i_ *I*_(ih)_.

^*d*^ *R*_work_ and *R*_free_ = ∑|*F*_obs_ – *F*_calc_| / ∑|*F*_obs_| × 100 for 95% of recorded data (*R*_work_) or 5% data (*R*_free_).

^*e*^ Determined using MolProbity.

**Fig 2:**
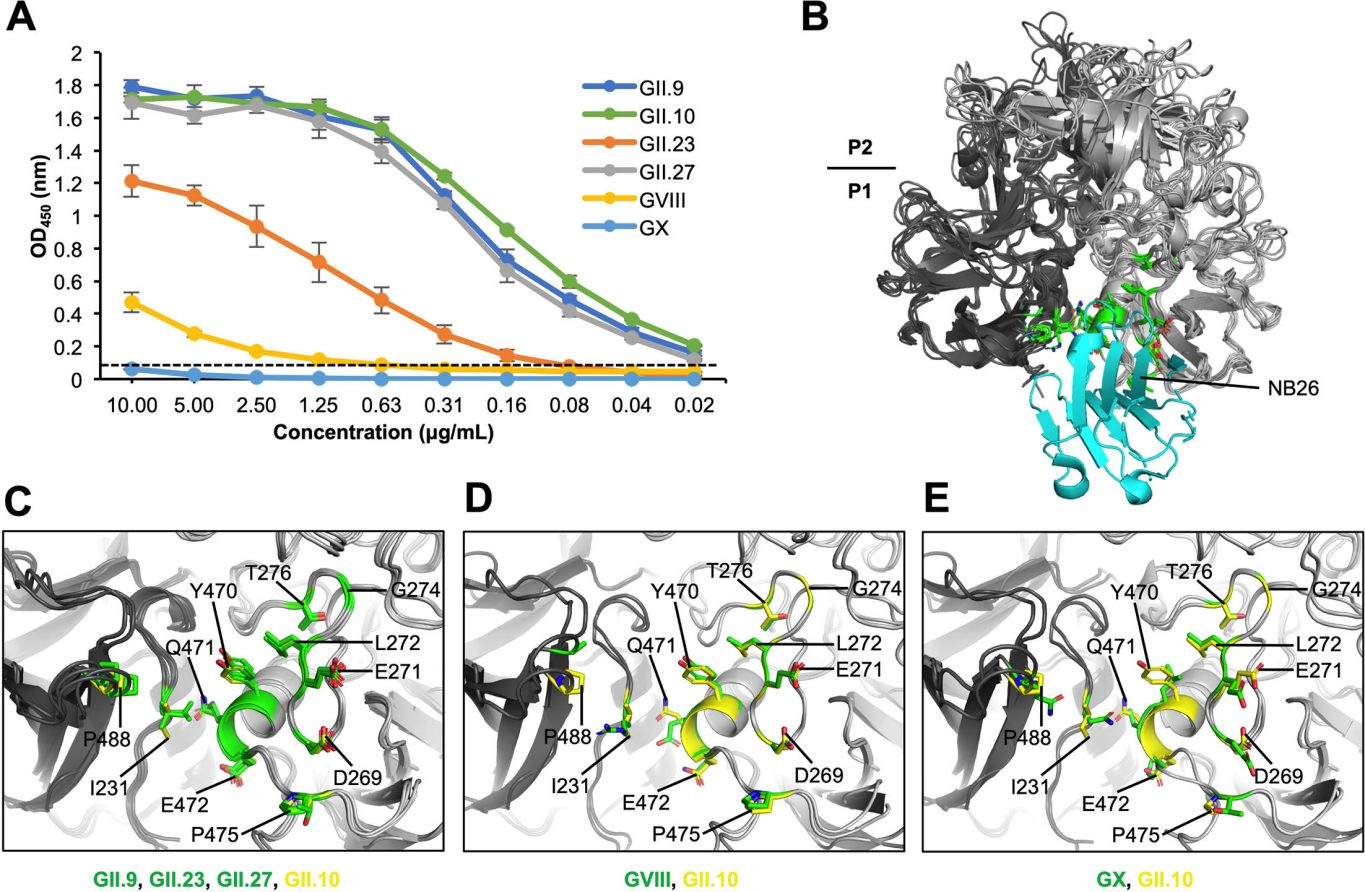
Fc-NB26 cross-reactivity and epitope binding characterization among these five P domains. (**A**) Cross-reactivity of Fc-NB26 to GII.9, GII.23, GII.27, GVIII, and GX P domains using direct ELISA with serial diluted Fc-NB26 from a starting dilution of 10 µg/mL in PBS. Error bars are shown (triplicate wells), and the dashed line represents the cutoff at an optical density of 450 (OD_450_) = 0.05 (15–17). (**B**) Superposition of GII.10 P domain NB26 complex (PDB ID: 5O04) and the five P domain structures (chain A and B colored light and dark gray, respectively). (**C**) Closeup of equivalent P domain residues (green) potentially interacting with Fc-NB26, where underlined residues show substitutions in GII.9 (chain A: D269, E271, L272, G274, T276, Y459, Q460, E461, T464, and chain B: V231 and P477), GII.23 (chain A: D269, E271, L272, G274, T276, Y459, Q460, E461, P470, and chain B: I231 and P483), GII.27 (chain A: D269, E271, L272, G274, T276, Y459, Q460, E461, P470, and chain B: I231 and P483), and with reference GII.10 (chain A: D269, E271, L272, G274, T276, Y470, Q471, E472, P475, and chain B: I231 and P488). The residue numbering refers to GII.10 P domain and the side chains (yellow) interacting with NB26. (**D**) Closeup of equivalent GVIII P domain residues (green) potentially interacting with Fc-NB26 (chain A: D269, E271, L272, G274, T276, Y476, E477, Q478, P481, and chain B: R231 and A494). (**E**) Closeup of GX P domain side chains (green) at the equivalent Fc-NB26 pocket shows numerous amino acid substitutions (underlined) that could restrict Fc-NB26 binding (chain A: E270, E272, L273, G275, T277, L455, T456, H457, T460, and chain B: N232 and Q473).

The cross-reactivity of the P domains was determined using direct ELISA with a broad-spectrum human norovirus Nanobody (Fc-NB26) that binds to a highly conserved region on the P domain (15, 16). Fc-NB26 bound to GII.9, GII.23, and GII.27 P domains in a dose-dependent manner at concentrations less than 80 ng/mL (Fig. 2A). Comparable binding values were observed with other human GII genotypes (GII.1, GII.4, GII.8, GII.10, GII.14, GII.17, GII.24, GII.26, and GII.NA1) (15, 16). Fc-NB26 bound to the GVIII P domain at weaker concentrations than these GII genotypes, while Fc-NB26 did not bind to the GX P domain at any concentration tested (Fig. 2A). Structural modeling of the Fc-NB26-binding site indicated that GII.9, GII.23, and GII.27 P domains contained most of the equivalent residues that could interact with Fc-NB26 (Fig. 2B and C) (15, 16). The GVIII P domain also had many Fc-NB26-binding residues (Fig. 2D), while most Fc-NB26-binding residues were absent in GX (Fig. 2E).

Our cumulative data show that Fc-NB26/NB26 binds most GII genotypes, and the epitope is vulnerable and a therapeutic target region for GII noroviruses (15, 17, 18). Subsequently, the bat norovirus could be considered antigenically distinct from human GII noroviruses. In addition, the bat norovirus HBGA-binding site may be shifted from the regular GII HBGA pocket, as observed in other genogroups (14, 16, 19–23).

## Data Availability

Coordinates and structure factors were deposited into the Protein Data Bank under the following ID numbers: GII.9-VA97207, PDB ID 9EDM; GII.23-Loreto1847, 9EDN; GII.27-Loreto0959, 9EDO; GVIII-Chiba040502, 9EDP; and GX-NPIH26, 9EDQ.
